# Prospective Randomized Double-Blind Placebo-Controlled Study of Oral Pomegranate Extract on Skin Wrinkles, Biophysical Features, and the Gut-Skin Axis

**DOI:** 10.3390/jcm11226724

**Published:** 2022-11-14

**Authors:** Mincy Chakkalakal, Dawnica Nadora, Nimrit Gahoonia, Ashley Dumont, Waqas Burney, Adrianne Pan, Cindy J. Chambers, Raja K. Sivamani

**Affiliations:** 1 Integrative Skin Science and Research, Sacramento, CA 95815, USA; 2 College of Medicine, California Northstate University, Elk Grove, CA 95757, USA; 3 Coastal Thyme Skin, Portsmouth, NH 03801, USA; 4 Pacific Skin Institute, Sacramento, CA 95815, USA; 5 Department of Dermatology, University of California-Davis, Sacramento, CA 95816, USA

**Keywords:** punicalagin, functional food, polyphenols, antioxidants, skin health, pomegranate, wrinkles, pomella, gut-skin axis, microbiome

## Abstract

(1) Background: The pomegranate fruit *(Punica granatum* L.) has been widely used in traditional medicine and has increasingly gained popularity among consumers in order to manage different facets of health. The objective of this study was to evaluate the effects of the fruit extract of *P. granatum* L. on different parameters of skin health. (2) Methods: A prospective, double-blind placebo-controlled study was conducted on both healthy males and females aged 25–55 years. Subjects were supplemented with a standardized punicalagin enriched oral pomegranate extract [Pomella^®^ (Verdure Science, Noblesville, IN, USA), PE group] or a placebo (control group) daily for four weeks. Changes in wrinkle severity, facial biophysical properties, skin microbiome, and the gut microbiome were assessed. (3) Results: The PE group had significant reductions in wrinkle severity (*p* < 0.01) and a decreasing trend in the forehead sebum excretion rate (*p* = 0.14). The participants in the PE group with a higher relative abundance of *Eggerthellaceae* in the gut had a decrease in their facial TEWL (*p* < 0.05) and wrinkle severity (*p* = 0.058). PE supplementation led to an increase in the *Staphylococcus epidermidis* species and the *Bacillus* genus on the skin. (4) Conclusions: Overall, the study demonstrated improvements in several biophysical properties, wrinkles, and shifts in the skin microbiome with oral PE supplementation in healthy subjects.

## 1. Introduction

Skin health and physical appearance has played an integral role throughout history and modern day society in defining overall health and well-being The skin has a myriad of functions; it directly impacts numerous systems of the body, ranging from acting as a barrier to external pathogens to providing temperature regulation [1]. In addition to many physical impacts on health, the appearance and quality of skin also has mental and cultural impacts. For example, psychologically, skin health can affect an individual’s sense of self-esteem, self-confidence, and quality of life [2]. Additionally, it is an important contributor to physical appearance and beauty in various cultures. For instance, certain skin factors, such as the appearance of wrinkles, are attributed to the level of an individual’s physical attractiveness and desirability [3]. Thus, one’s skin impacts physical, social, and psychological aspects of health.

For these many reasons, continually developing new strategies to maintain skin health and appearance is of great importance. Current methods include, but are not limited to, topical cosmetic products, systemic therapeutic agents, and non-invasive procedures. In recent years, preventative measures, such as diet and lifestyle changes, have emerged as effective strategies for the maintenance of skin health. For example, investigators reported that consuming a balanced diet rich in antioxidants is a key factor in reducing the harmful effects of free radicals in our system [4]. This is of great relevance as an excess of reactive oxygen species is implicated in photodamage, photoaging, and even some skin cancers. As a result, incorporating these antioxidant agents through nutritional supplements and functional foods has gained popularity within the field of dermatology in maintaining overall healthy skin appearance [3].

Pomegranate fruits are widely consumed as a food, as both edible arils and squeezed fruit juice, but also as extracts as oral dietary supplements [5,6,7]. Pomegranate and its different parts are rich in more than 100 bioactive constituents, including ellagitannins, gallotannins, anthocyanins, flavanol–anthocyanin adducts, flavonoids, phenolic acids and other phenols. The pomegranate contains different classes of polyphenols including ellagitannins, which are hydrolyzable tannins subsuming punicalagin, punicalin, and pedunculagin [5,8,9]. The antioxidant, anti-inflammatory, anti-microbial, anti-neuroinflammatory, anti-glycation, and immunomodulatory properties of the different components of the pomegranate fruit have been well-elucidated [9,10,11,12,13,14,15]. For example, the pomegranate’s antioxidant capacity was reported to be the highest among several dietary nutritional supplements including milk thistle, green tea, grape seed, gogi, and acai extracts [16]. Other studies have also reported that the phytochemicals found within the pomegranate confer protection against UVA and UVB-mediated cell damage in human skin fibroblasts in-vitro and reduce the oxidative stress, DNA damage, and cytotoxicity induced by harmful agents such as hydrogen peroxide and methylglyoxal in human keratinocytes in-vitro [11,12,17]. Additionally, the positive health effects of pomegranate on liver health, obesity, and the gut microbiome have also been established [18].

Antioxidants, both natural and synthetic, are increasingly used as functional food additives and nutritional supplements. Oxidative reactions can damage proteins, lipids, and DNA due to an uneven ratio of antioxidants to free radicals. The capacity of antioxidants to stop oxidative deterioration in food and pharmaceutical items, as well as in the body and against disease processes brought on by oxidative stress, has heightened interest in them. Numerous examinations exploring the connection between food and human health have been carried out using diverse study focus samples [19]. In this double-blind, placebo-controlled clinical trial, we aimed to understand the effects of oral supplementation of a standardized pomegranate fruit extract (Pomella^®^, Verdure Science, Noblesville, IN, USA) on the facial appearance of wrinkles and on different biophysical properties of the skin, including facial sebum production and transepidermal water loss. The study also involved an assessment of the skin microbiome as well as the role of the gut microbiome in modulating the results.

## 2. Materials and Methods

### 2.1. Study Design, Recruitment, and Randomization

This study was conducted between November 2020 and March 2021 as a four-week double-blind, placebo-controlled pilot clinical trial. This study was conducted with the approval of the Institutional Review Board (IntegReview Ltd., Austin, TX, USA) and registered at www.clinicaltrials.gov (NCT04596722, accessed on 8 November 2022). All participants provided written informed consent prior to participation in the study. Males and females living in the Sacramento region, ages 25–55 were recruited from a local dermatology clinic through the use of social media advertisements. Individuals were screened for eligibility and all study procedures were performed at Integrative Skin Science and Research in Sacramento, CA, USA.

**Figure 1:**
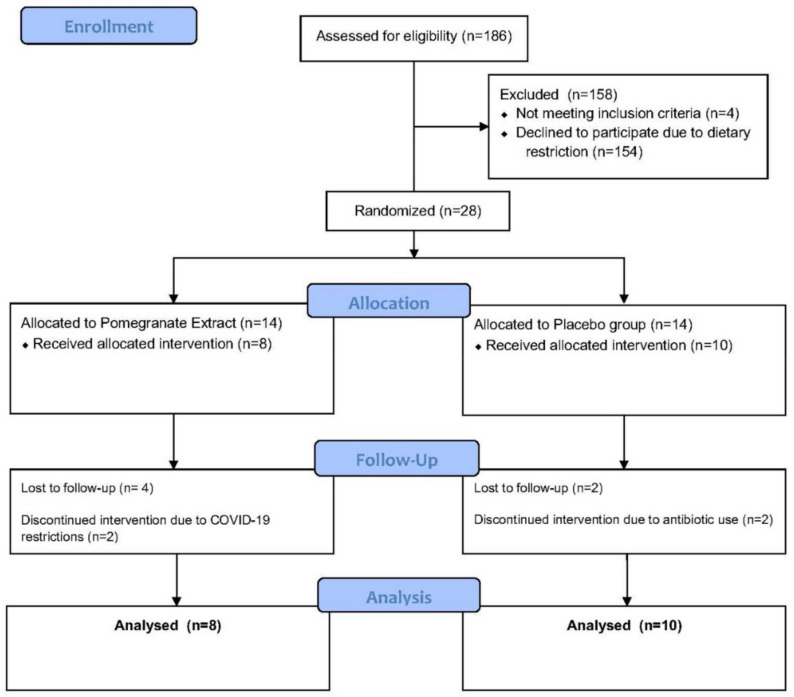
Consort Flow Diagram outlining the design of the randomized study evaluating the pomegranate extract compared against placebo.

In this case, 186 individuals were assessed for eligibility, of which 154 declined to participate due to the dietary restrictions required by the study. In addition, 4 individuals failed to meet the inclusion criteria. Here, 28 subjects met inclusion criteria, and were randomized and pre-allocated into the following two study groups using a computer-based randomization generator with blinded sealed envelopes. The PE group (n = 14) received an oral supplement (Pomella^®^, Verdure Sciences, Inc. Noblesville, IN, USA) containing 75 mg of punicalagin per capsule and the control group (n = 14) received an oral placebo for daily consumption for four weeks. In the control group, 2 subjects were lost to follow-up after screening and 2 discontinued the study intervention due to antibiotic use. In the PE group, 4 subjects were lost to follow-up after screening and 2 discontinued the study intervention due to concerns related to COVID-19. This information is depicted in Figure 1.

### 2.2. Inclusion and Exclusion Criteria

Individuals with a known allergy to the study agents were not included in the study. The subjects were asked to discontinue the use of other nutritional supplements, including prebiotics and probiotics, one month prior to the baseline visit and throughout the study. The use of topical antibiotics and benzoyl peroxide were not permitted one month prior to the baseline visit and throughout the study. Individuals who had used systemic, injected, or oral antibiotics within 6 months of the baseline visit or those who had started a new diet such as the ketogenic diet within one month of the baseline visit were excluded from the study. Current smokers, individuals who have smoked within the past one year or with a five-year pack history of smoking tobacco were excluded from the study. Individuals with a history of the following conditions were also screened out of the study: malignancy, cancer (excluding non-metastatic cancer), gastrointestinal inflammatory diseases, epilepsy, immunologic or infectious diseases such as hepatitis, tuberculosis, human immunodeficiency virus, acquired immunodeficiency syndrome, lupus, or rheumatoid arthritis. Other exclusion criteria included the current use of medications that alter blood lipids, such as statins and anti-hyperlipidemic medications, individuals with a body mass index higher than 35 kg/m², individuals who adhere to the vegan diet, individuals who refuse to shave facial hair which may interfere with image collection and assessment, individuals who are participating in or had participated in an intervention based facial study two weeks prior to the baseline visit, women who have been pregnant in the last three months, are currently pregnant, preparing to be pregnant or lactating, and prisoners and adults who are unable to provide consent on their own.

The subjects enrolled in the study were also asked to adhere to the following set of dietary restrictions to reduce the confounding effects of polyphenolic-rich foods. The subjects were asked to reduce coffee intake to at most to an eight-ounce cup of coffee, a cup of berries, and a cup of fermented dairy products per week throughout the duration of the study. This diet also included the complete restriction of chocolate, alcohol, tea, and pomegranate or pomegranate-containing drinks for the four weeks of the study. Subjects were asked to completely avoid self-tanning, spa tanning, sun tanning or artificial tanning, dry or wet sauna treatments, and swimming. This was carried out to reduce the confounding effects of environmental or exogenous agents on the appearance and health of the skin.

### 2.3. Facial Imaging, Measurements of the Biophysical Properties of the Skin and Subjective Assessments of Digestive Health and Quality of Life

All measurements were collected after subjects had adjusted to ambient conditions for 15 minutes in a climate-controlled room. The following measurements were collected at the initial baseline visit and after four weeks of oral supplementation.

The facial appearance of wrinkles was assessed using high-resolution facial photographs captured and analyzed by the BTBP 3D Clarity Pro^®^ Facial Modeling and Analysis System (Brigh-Tex BioPhotonics, San Jose, CA, USA). The “average severity” of wrinkles was calculated by measuring the depth and width of the wrinkles.

The following biophysical properties of the skin on the forehead and cheeks were measured: facial transepidermal water loss (TEWL) using the Vapometer^®^ (Delfin Technologies Ltd., Kuopio, Finland) and facial sebum production by the Sebummeter^®^ SM 815 (Courage and Khazaka, Cologne, Germany). The SkinColorCatch^®^ (Delfin Technologies Ltd., Kuopio, Finland) was used to measure the facial erythema index and melanin index of the skin. The devices used to assess the biophysical properties of the skin are non-invasive instruments that contact the skin for, at, most 30 s.

Self-assessments of Quality of Life and Digestive Health were collected.

### 2.4. Skin Swab Collection, Stool Collection and DNA Extraction

Stool samples were collected from subjects at baseline and 4 weeks using a manually assembled kit for gut microbiome sampling. Facial skin swabs were collected from the glabella and cheeks at baseline and 4 weeks using kits from CosmosID^®^ (Germantown, MD, USA). Swabbing was performed using a sterile technique and samples were collected from the ambient environment to serve as negative controls during skin microbiome analysis. All samples were stored at −80 degrees Celsius prior to shipping.

Taxonomic and functional analyses of the 16S skin samples and WGS stool samples were performed by CosmosID^®^. The DNA from skin and fecal samples was isolated using the QIAGEN DNeasy PowerSoil Pro Kit, according to the manufacturer’s protocol. Extracted DNA samples were quantified using Qubit 4 fluorometer and Qubit™ dsDNA HS Assay Kit (Thermofisher Scientific, Waltham, MA, USA).

#### 2.4.1. Library Preparation and Whole Genome Sequencing of Stool Samples

DNA libraries were prepared using the Nextera XT DNA Library Preparation Kit (Illumina, San Diego, CA, USA) and IDT Unique Dual Indexes with total DNA input of 1 ng. Genomic DNA was fragmented using a proportional amount of Illumina Nextera XT fragmentation enzyme. Unique dual indexes were added to each sample followed by 12 cycles of PCR to construct libraries. The DNA libraries were purified using AMpure magnetic Beads (Beckman Coulter, Brea, CA, USA) and eluted in QIAGEN EB buffer. In addition, the DNA libraries were quantified using Qubit 4 fluorometer and Qubit™ dsDNA HS Assay Kit (Invitrogen, Waltham, MA, USA). The libraries were then sequenced on an Illumina HiSeq 4000 platform 2 × 150 bp.

#### 2.4.2. Bioinformatics Analysis of Samples

Unassembled sequencing reads were directly analyzed by CosmosID-HUB Microbiome Platform (CosmosID Inc., Germantown, MD, USA) as described elsewhere for multi-kingdom microbiome analysis and the profiling of antibiotic resistance and virulence genes, and quantification of organisms’ relative abundance [20,21,22,23]. Briefly, the system utilizes curated genome databases and a high-performance data-mining algorithm that rapidly disambiguates hundreds of millions of metagenomic sequence reads into the discrete microorganisms engendering the particular sequences. Similarly, the community resistome and virulome, the collection of antibiotic resistance and virulence-associated genes in the microbiome, were also identified by querying the unassembled sequence reads against the CosmosID curated antibiotic resistance and virulence-associated gene databases.

### 2.5. Statistical Analysis

All parametric data results are presented as the mean ± standard deviation. All non-parametric data results are presented as the median ± 95% confidence interval. The statistical analysis for parametric was performed using Student’s *t*-test to assess the within-group (two tailed, paired) and between group (two tailed, unpaired) differences. A chi-square test with Yates’ correction was performed to analyze non-parametric data. Yates’s correction was applied to reduce the overestimation of statistical significance for the survey results, which was composed of responses that had an expected count smaller than five. Any values of (*p* < 0.05) were considered statistically significant. Each subject served as their own control, as values reported at four weeks were compared to baseline values, or 1. All data were analyzed including subjects that were enrolled in the study and those who had received any study intervention.

Differential microbiome abundance was determined using Linear discriminant analysis effect size (LEfse) analysis at all taxonomic levels. Differential feature and organism abundance between groups was calculated using Kruskal-Wallis sum-rank test, Wilcoxon rank-sum test, and Linear Discriminant Analysis. All features shown meet *p* ≤ 0.05 for Kruskal-Wallis and Wilcoxon tests and have an LDA score where effect−size ≥ 2.0. The statistical significance was determined using the Wilcoxon Rank-Sum Test. *p* < 0.05 was considered statistically significant.

## 3. Results

In this case, 15 females and 3 males completed the study. In the control group, there were 10 participants (F = 9, M = 1). The average age of participants in this group was 42.3 ± 10.5 and the average BMI was 24.4 ± 4.5. In the PE group, there were 8 participants (F = 6, M = 2). The average age of the participants in this group was 40 ± 11.2 and the average BMI was 24.6 ± 4.6.

### 3.1. Facial Wrinkle Severity

**Figure 2:**
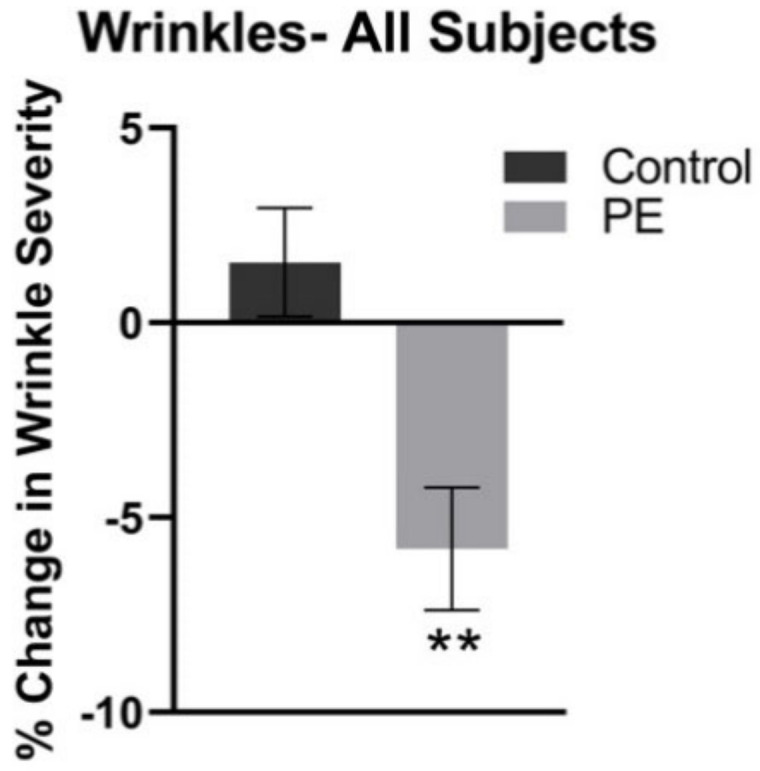
Mean Facial Wrinkle Severity in the Control and PE Group, Relative to Pretreatment Baseline. The facial wrinkle severity was measured through the use of high-resolution imaging analysis of standardized photography. The average facial wrinkle severity decreased by 6.2 ± 1.6 (** *p* = 0.004) in the PE group and there was no statistical change in the control (placebo) group.

There was a statistically significant decrease in the facial wrinkle severity by 6.2 ± 1.6% in the PE group *(p <* 0.01) compared to 1 ± 1.4% increase in the control group. These results are demonstrated in Figure 2.

### 3.2. Facial Sebum Production

**Figure 3:**
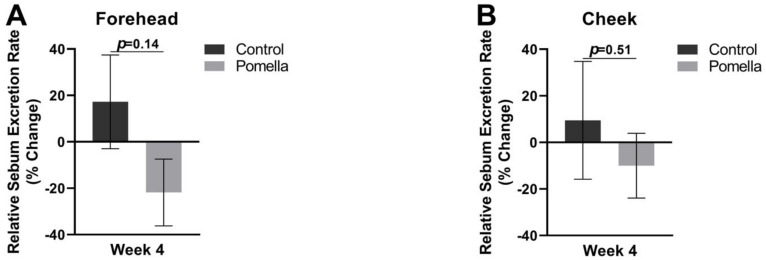
The sebum excretion rate in the Control and PE groups relative to pre-treatment baseline. Sebum was measured with a sebumeter and week 4 measures were compared against baseline on the forehead (**A**) and on the cheek (**B**).

There was a decreasing trend in the sebum excretion rate on the forehead of those in the PE supplementation group after four weeks with a 21.9 ± 14.4% reduction compared to an increase of 17.2 ± 20.2% (*p* = 0.14). There was no statistically significant difference in the cheek sebum excretion rate between subjects in the control group and PE group, as compared to the pretreatment baseline. The results are demonstrated in Figure 3.

### 3.3. Facial TEWL

**Figure 4:**
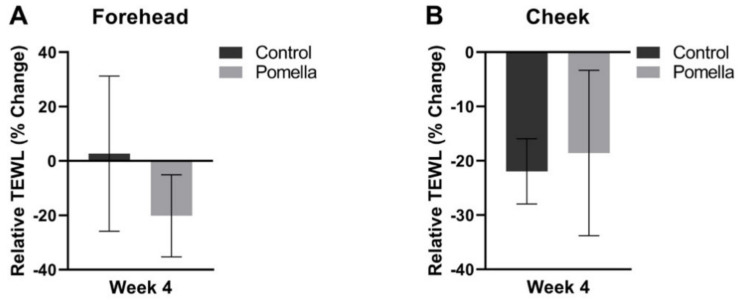
Transepidermal water loss (TEWL) in the Control and PE groups relative to pre-treatment baseline. The TEWL at 4 weeks was compared against baseline on the forehead (**A**) and the cheeks (**B**).

When compared to pretreatment baseline, the observed differences in the forehead and cheek TEWL between subjects in the control and PE group were not statistically significant (Figure 4). Interestingly, in the PE group subjects with a higher level of *Eggerthellaceae* in their gut microbiome analysis showed a statistically significant reduction in the TEWL compared to those that did not express *Eggerthellaceae*.

### 3.4. Skin Colormetric Indices (Melanin and Erythema)

There were no statistically significant changes in the facial melanin or erythema indices between the PE and control groups.

**Figure 5:**
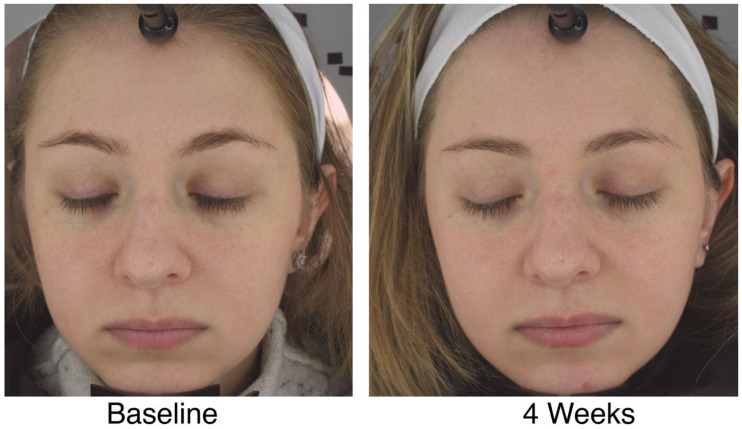
Facial images of a study participant at baseline and week 4 from the PE group.

Figure 5 shows a typical participant before and after intervention from the PE group.

### 3.5. Influence of the Gut Expression of Eggerthellaceae in the PE Group

**Figure 6:**
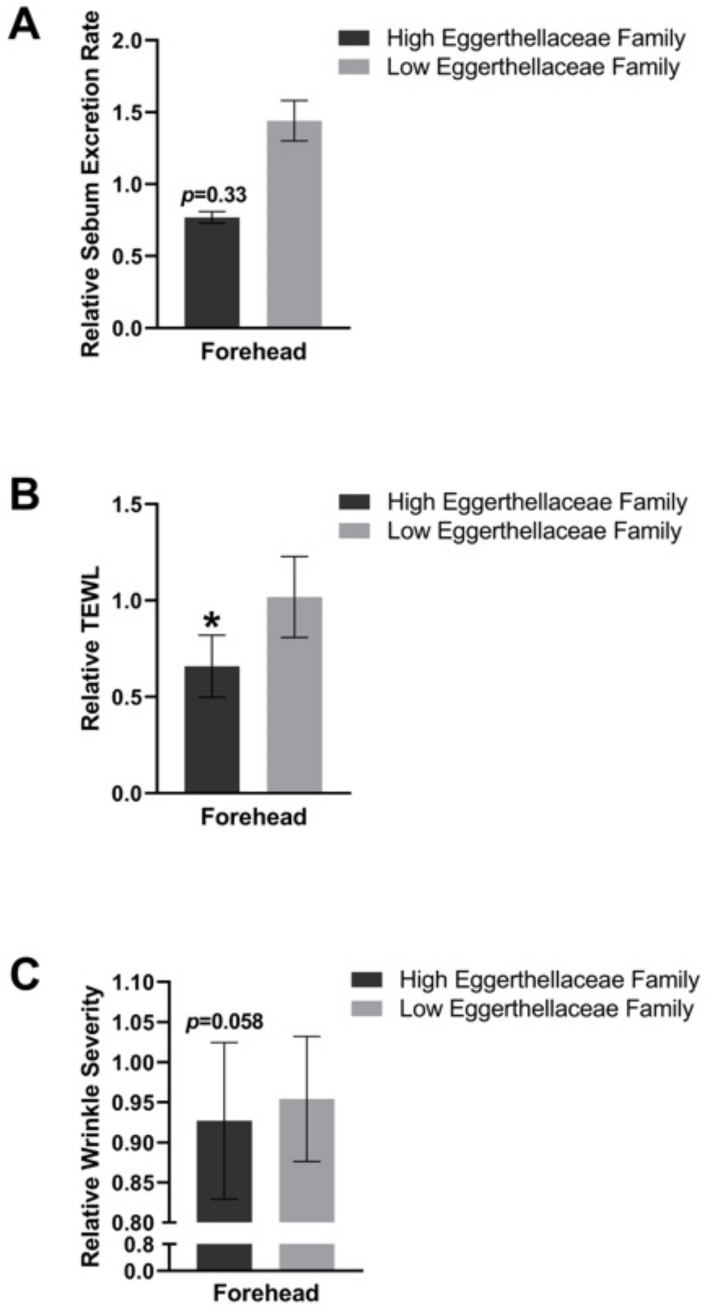
Sub-analysis based on high expression of *Eggerthellaceae* in the gut. The participants in the PE group were stratified as high or low expression of *Eggerthellaceae* in the gut and the influence on sebum excretion rate (**A**), TEWL (**B**), and wrinkle severity (**C**) are depicted. * = *p* < 0.05.

A sub-analysis among participants in the PE group for the gut microbiome expression *Eggerthellaceae* was performed. A relative decrease in sebum was seen in those that had a higher expression of *Eggerthellaceae*, but this was not statistically significant (*p* = 0.33). Interestingly, the higher expression of *Eggerthellaceae* in the gut microbiome of study participants was found to correlate with a decrease in TEWL (*p* < 0.05) and wrinkle severity (*p* = 0.058). These results are depicted in Figure 6.

### 3.6. Shifts in the Skin Microbiome

**Figure 7:**
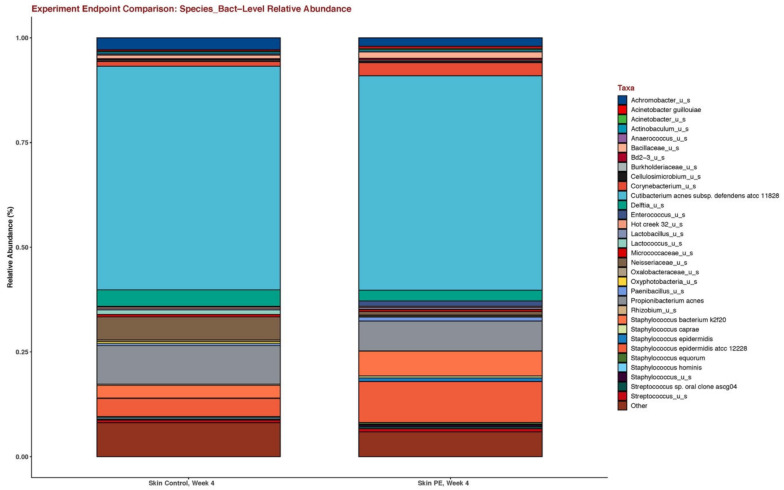
Stacked bar plots of the skin microbiome after 4 weeks of supplementation in the placebo and PE group. The skin microbiome was collected through the use of facial skin swabs.

**Figure 8:**
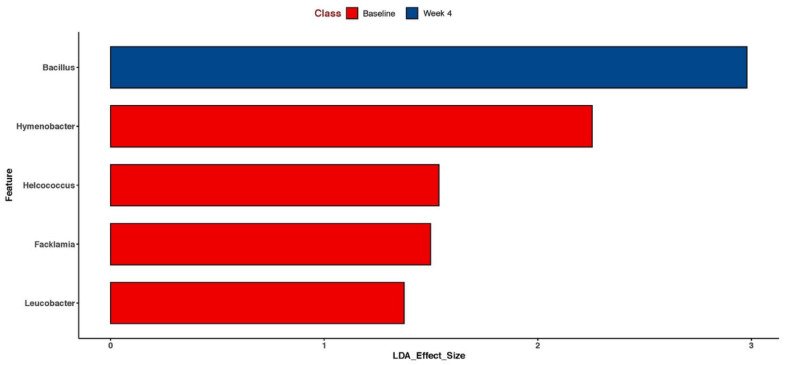
Lefse analysis of the skin microbiome in the PE group before (pretreatment) and after supplementation (4 weeks of PE) shows enrichment for the *Bacillus* genus. Only statistically significant results are shown.

The skin microbiome was shifted in those in the PE group with an increase in the relative abundance of the *Staphylococcus epidermidis* species (Figure 7) and the *Bacillus* genus (Figure 8).

### 3.7. Adverse Events

No adverse events were reported throughout the study.

## 4. Discussion

In this 4-week, randomized, double-blind, placebo-controlled study, supplementation with 250 mg of a standardized pomegranate fruit extract containing 75 mg punicalagin (Pomella^®^) once a day showed a significant decrease in image-based facial wrinkle severity and a decreasing trend in facial sebum excretion rate compared to placebos. Furthermore, the higher levels of *Eggerthellaceae* in the gut microbiome were correlated with a decrease in both TEWL and wrinkle severity in the PE group, suggesting that the extract improved skin barrier function in addition to improving the appearance of wrinkles.

The disruption in skin homeostasis due to increased formation of advanced glycation end-products, oxidative stress and other intrinsic and extrinsic factors resulted in the loss of skin barrier function. This is further associated with the thinning of the epidermis and the subsequent appearance of deeper and more spaced-out wrinkles [24]. The mechanism of action for the decrease in wrinkles is not clear but PE is rich in antioxidants and may be protective against collagen breakdown. Moreover, our subanalysis among the PE group for those that expressed *Eggerthellaceae* more abundantly showed a correlation for a decrease in both TEWL and facial wrinkle severity. The restoration of the skin barrier may result in an improvement in TEWL and the appearance of wrinkles. Therefore, improvement in the skin barrier function of subjects in the PE group due to a reduction in the TEWL may be partially responsible for the significant reduction in facial wrinkle severity observed within the same cohort.

Berardesca et al. and Grubauer et al. reported that decreases in TEWL from the stratum corneum of the epidermis is associated with enhanced skin barrier function and aged skin [24,25]. Furthermore, Lin et al. suggest that although an inflammatory response and the subsequent production of reactive oxygen species and secretion of cytokines is necessary for skin barrier repair, excessive inflammation results in the disruption of skin homeostasis and the reduction of skin barrier function [26]. It is important to note that many studies have provided compelling evidence for the antioxidant and anti-inflammatory activity associated with the phytochemicals of the fruit *P. granatum* L., which constitute the oral PE utilized in the study [8,27,28,29,30,31].

The significant decrease in wrinkle severity among the subjects in the PE group may also be supported by a study that reported that pomegranate concentrated solution (PCS) possesses anti-wrinkle effects due to its ability to decrease elastase and collagenase activities in normal human primary dermal fibroblast-neonatal (HDF-N cells) [32].

It appears that previous studies of PE extracts are in agreement with the findings reported here. A 2012 study demonstrated that a 55-day pomegranate flower extract supplementation restored the decrease in skin water content and epidermal barrier function after inducing disruption in skin homeostasis through a tape-stripping procedure in rodent models [33]. Notably, the flowers of the pomegranate contain phenolic acids such as gallic acid and ellagic acid, which are both found in the pomegranate peel. However, the flowers are unique in that they contain triterpenoids, such as oleanolic acid and ursolic acid. Since the investigators of this study did not attribute the study findings to specific phytoconstituents, future studies should evaluate whether the observed results were due to the effects of the phenolic acids, triterpenoids, or both of the bioactive compounds. Since the pomegranate peel shares similar phytoconstituents with the pomegranate flower, it is not surprising that there were changes found with TEWL in this clinical study.

The trend noted for the decrease in the sebum excretion rate in the PE group and in those that highly expressed *Eggerthellaceae* in the gut has mechanistic support from previous studies. A 2017 study reported the anti-lipase activity of PE and specific tannins isolated from the fruit of *P. granatum* L. such as punicalagin and punicalin. Specifically, PE was found to reduce lipase activity by 20%, whereas punicalagin and punicalin reduced lipase activity by 39.8% and 34.7%, respectively. These results are singificant, as the overproduction of sebum is associated with increased bacterial lipase activity in some skin conditions such as acne vulgaris. Since the PE utilized in our study contained 75 mg of punicalagin, our inhibitory findings on facial sebum production are consistent. In addition, Lee at al. studied the topical application of PE and its isolated tannins in rodent models [34] in further support of the findings noted here. In light of the sebum modulation of PE, future studies should consider the study of PE supplementation in acne where excess sebum production is a notable feature.

We find it interesting that we were able to stratify responses based on the gut expression of *Eggerthellaceae.* Bacterial species belonging to *Eggerthellaceae* are able to produce urolithin metabolites from ellagic acid and ellagitannins, which are found in pomegranates and enriched in the PE used in this study [35]. Therefore, our results suggest that the presence of *Eggerthellaceae* may predict a better response to PE supplementation and support the notion of a gut-skin axis when considering oral supplementation.

Our skin microbiome studies showed an augmentation in *Staphylococcus epidermidis* and in the *Bacillus* species. The role of the shift in *S. epidermidis* is unclear at this time. In atopic dermatitis, an increase in the *S. epidermidis* relative abundance is correlated with an improved skin barrier but in seborrheic dermatitis, an increase in *S. epidermidis* is correlated with an impaired skin barrier [36,37]. The role of the *Bacillus* species is less clear, although ferments and lysates of *Bacillus coagulans* have been shown to have anti-inflammatory and immune-modulating effects and are currently in use in the cosmetics market [38,39]. Nevertheless, our study highlights that oral supplementation with PE alters the skin microbiome.

Our study has several limitations. This study was conducted over four weeks and limits our ability to detect large shifts in the appearance of wrinkles. However, we utilized validated high-resolution imaging that allows us to detect smaller shifts in a reproducible and objective fashion. Another limitation is that we utilized multiple food-related exclusions, such as limiting the intake of tea and coffee. While this increases the rigor of the study, it may not mimic the real-life scenario for many people who likely ingest tea and coffee regularly. Finally, this study serves as a pilot study, and further studies with an expanded cohort are warranted.

## 5. Conclusions

Oral supplementation with 250mg of Pomella^®^ pomegranate fruit extract standardized to 75 mg of punicalagins daily for four weeks significantly improved several parameters of skin health. Specifically, our data demonstrate reductions in the appearance of average facial wrinkle severity, a trend for decreasing forehead sebum excretion rate, a shift in the skin microbiome, and accentuated responses for TEWL reduction and wrinkle reduction in those that express *Eggerthellaceae* in their gut. These effects may be related to the potent antioxidant and anti-inflammatory properties of the phytochemicals, which constitute the oral interventional agent. Future studies with an expanded population are needed in order to evaluate the inhibitory trend we observed in facial sebum production within the PE cohort.
